# Influence of Different Three-Dimensional Open Porous Titanium Scaffold Designs on Human Osteoblasts Behavior in Static and Dynamic Cell Investigations

**DOI:** 10.3390/ma8085259

**Published:** 2015-08-24

**Authors:** Jana Markhoff, Jan Wieding, Volker Weissmann, Juliane Pasold, Anika Jonitz-Heincke, Rainer Bader

**Affiliations:** 1 University Medicine Rostock, Department of Orthopaedics, Biomechanics and Implant Technology Laboratory, Doberaner Strasse 142, Rostock 18057, Germany; E-Mails: jan.wieding@med.uni-rostock.de (J.W.); juliane.pasold@med.uni-rostock.de (J.P.); anika.jonitz@med.uni-rostock.de (A.J.-H.); rainer.bader@med.uni-rostock.de (R.B.); 2 Institute for Polymer Technology, Alter Holzhafen 19, Wismar 23966, Germany; E-Mail: weissmann@ipt-wismar.de

**Keywords:** scaffold, Ti-6Al-4V, microstructure, bioreactor, human osteoblasts, static culture, dynamic culture, additive manufacturing

## Abstract

In the treatment of osseous defects micro-structured three-dimensional materials for bone replacement serve as leading structure for cell migration, proliferation and bone formation. The scaffold design and culture conditions are crucial for the limited diffusion distance of nutrients and oxygen. In static culture, decreased cell activity and irregular distribution occur within the scaffold. Dynamic conditions entail physical stimulation and constant medium perfusion imitating physiological nutrient supply and metabolite disposal. Therefore, we investigated the influence of different scaffold configurations and cultivation methods on human osteoblasts. Cells were seeded on three-dimensional porous Ti-6Al-4V scaffolds manufactured with selective laser melting (SLM) or electron beam melting (EBM) varying in porosity, pore size and basic structure (cubic, diagonal, pyramidal) and cultured under static and dynamic conditions. Cell viability, migration and matrix production were examined via mitochondrial activity assay, fluorescence staining and ELISA. All scaffolds showed an increasing cell activity and matrix production under static conditions over time. Expectations about the dynamic culture were only partially fulfilled, since it enabled proliferation alike the static one and enhanced cell migration. Overall, the SLM manufactured scaffold with the highest porosity, small pore size and pyramidal basic structure proved to be the most suitable structure for cell proliferation and migration.

## 1. Introduction

The application of synthetic, micro-structured three-dimensional scaffolds for the treatment of large bone defects has become an adequate alternative to bone autografts and allografts with their limited availability and risk of infection or rejection [1,2]. Due to good mechanical properties and excellent biocompatibility resulting from a formed oxide layer, metallic materials, e.g., Ti-6Al-4V, are mainly used in orthopedic surgery [3,4].

Porous metallic materials can exhibit compression strength similar to cortical bone [5] and are, in contrast to ceramics and polymers, applicable for load-bearing areas [6]. Metallic materials are inserted in form of titanium foams, [4,7] fiber meshes [8,9] or open-porous scaffolds with regular structure [1,5,6,10]. Thereby, the cell growth is influenced by the roughness of surfaces [3,11] and the structure of pores, *i.e.*, pore size, shape, porosity and interconnectivity [12]. These requirements can be controlled by the manufacturing methods. Fabrication of porous three-dimensional structures based on metal and alloy is hindered by reasons of their chemical reactivity and high melting/sintering temperatures [13]. Techniques like plasma-spraying, sintering, shot-blasting or acid-etching [9] only allow limited control over the pore structure on the scaffold surface [14,15]. As against conventional material forming processes, the lower-cost and faster additive-manufacturing processes, e.g., laser- or electron-beam-melting [1,16] permit gradual-building of solid metallic material from powder [17] and enable a controllable microstructure; an interconnective porosity with defined pore size [18,19]. By modifying the micro-structure of these porous constructs an adjustment of the mechanical properties is possible; e.g., similar to these of bone [19]. However, the scaffold’s porosity determines the permeability [20], but also the mechanical properties and is therefore limited [12]. For optimal cell growth, pore sizes in the range of mainly 50 to 500 µm [3,14,21,22] and an interconnective porosity of 60% to 75% [1,23] are mentioned. These parameters are crucial for cell seeding, migration, nutrient transport, matrix deposition and vascularization [3]. On the one hand, smaller pores result in increased proliferation due to a greater material surface for adhesion and growth [12] and also enable cell bridging over the pores [24] as well as an efficient cell seeding [18] considering that pore size is cell specific [25]. On the other hand, small pores limit scaffold permeability and thus, nutrient and waste exchange [18] as well as cell migration into the scaffold and vascularization [20].

Besides structural properties, scaffold colonization *in vitro* is determined by cultivation conditions. In static culture, cells on porous scaffolds are submitted to limited nutrient and oxygen supply as well as waste removal in the centre [26,27]. The resulting hypoxia decreases matrix production and cell viability and causes cell necrosis [28,29]. Furthermore, cells mainly adhere and proliferate at the outer face of the scaffold and tend to obstruct the upper pores, which avoids cell migration and diffusion processes into the scaffold [4]. To solve this problem, dynamic cultivation in bioreactors, e.g., spinner flasks, rotating wall vessels or cylindrical or vertical perfusion systems is used [30,31]. By applying a constant fluid flow *in vitro*, shear stress is carried out to the cells and mechanotransduction is initiated [32], so physical signals are converted into biochemical ones [33]. Increased cell proliferation, differentiation, migration, uniform cell distribution and matrix formation are proved in consequence of dynamic cultivation [34,35,36], which is due to enhanced nutrient and oxygen supply and waste removal [8]. Nevertheless, high flow rates can also lead to increased cell detachment or damage [35].

The aim of the present *in vitro* study was to compare the behavior of human osteoblasts on three-dimensional porous Ti-6Al-4V scaffolds of two manufacturing methods with different structural design features including pore size, porosity and basic structure under static and dynamic cultivation conditions.

## 2. Materials and Methods

### 2.1. Generation and Fabrication of Micro-Structured Titanium Scaffolds

**Figure 1:**
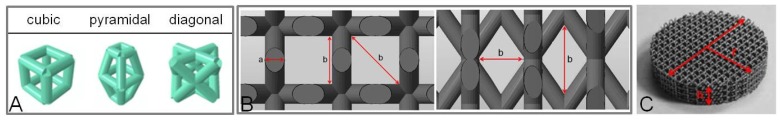
(**A**) Basic structures of the generated Ti-6Al-4V scaffolds (cubic, pyramidal and diagonal); (**B**) Span of strut thickness (a) and pore size (b) in µm exemplary for the cubic (left) and diagonal scaffold (right); (**C**) Manufactured Ti-6Al-4V scaffold with pyramidal basic structure.

The choice of structures and scaffold parameters was preceded by previous biomechanical studies [5,37]. Three scaffold designs were generated by means of CAD software (SolidWorks, SolidWorks Corporation, Concord, MA, USA) differing in their basic structure (Figure 1) as well as in porosity, pore size and strut design. The geometrical parameters of the scaffolds are constituted in Table 1. The cubic structure is characterized by rectangular pores in vertical orientation, while the pyramidal basic structure exhibited trapezoidal pores in both directions of the z-axis. Another basic structure is marked by diagonally-orientated struts and checked-like pores [5].

**Table 1:** Geometrical parameters of the generated Ti-6Al-4V scaffolds.

	Basic Structure			Scaffold Dimensions			
Scaffold Design	Strut Thickness ^a^ [µm]	Strut Cross-Sectional Area [mm^2^]	Pore Size ^b^ [µm]	Radius ^r^ [mm]	Height ^h^ [mm]	Surface Area [mm^2^]	Porosity [%]
cubic	700 *	0.490	700 × 700	17.5	5.5	~12200	~51
pyramidal	400 **	0.126	400–620	17	9.4	~18000	~76
diagonal	400 **	0.126	400–1000	17	10.4	~20800	~75

Notes: * struts exhibited a rectangular cross-section; ** struts exhibited a circular cross-section. Elevated letters are associated with Figure 1B,C.

**Figure 2:**
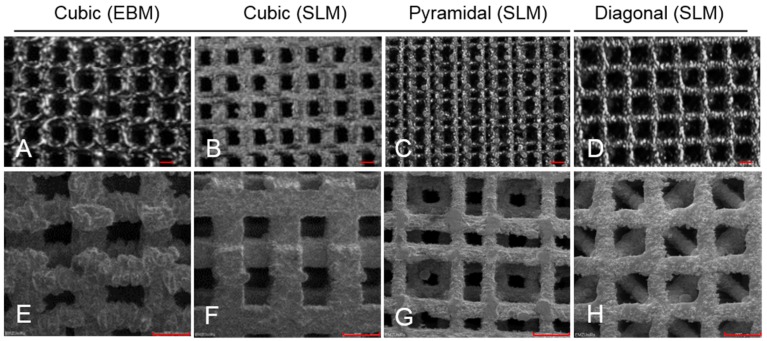
Light microscope (**A**–**D**; scale bar: 700 µm) and scanning electron microscope pictures (**E**–**H**; scale bar: 900 µm) of the Ti-6Al-4V scaffolds with four basic structures and fabricated with two different processes (EBM: electron beam melting; SLM: selective laser melting).

The scaffolds were fabricated of titanium powder (Ti-6Al-4V) using two different additive manufacturing processes (AM), selective laser melting (SLM) (SLM solutions GmbH, Luebeck, Germany) as well as electron-beam melting (EBM) (Institute for Materials Science, University of Erlangen-Nuremberg, Erlangen, Germany) (Figure 2). Thereby, the scaffolds are created in an inert gas or vacuum atmosphere by layerwise melting of the loose powder particles with a layer thickness of approximately 40 µm, depending on the process parameters [17].

### 2.2. Isolation and Cultivation of Human Primary Osteoblasts

Isolation of human primary osteoblasts followed the procedure described previously by Lochner *et al.* [38]. Briefly, cells were isolated from the spongiosa of the femoral heads of patients undergoing primary total hip replacement. The samples were collected with patient agreement and approval by the Local Ethical Committee (registration number: A 2010-10). Cultivation was done in osteogenic cell culture medium (MEM Dulbecco, Biochrom AG, Berlin, Germany) with 10% FCS, 1% penicillin/streptomycin, 1% amphotericin B, 1% HEPES buffer (all: Gibco^®^-Invitrogen, Darmstadt, Germany) including osteogenic additives (dexamethason (100 mM), L-ascorbic acid (50 mg/mL) and β-glycerophosphate (10 mM) (all from Sigma-Aldrich, Munich, Germany)). Alkaline phosphatase staining with fuchsin substrate chromogen (DAKO, Hamburg, Germany) was done to verify the osteogenic character of the isolated cells. Cultivation was carried out in an incubator (Binder GmbH, Tuttlingen, Germany) at simulated *in vivo* conditions 37 °C, 5% CO_2_ and 21% O_2_ for one week.

A total of seventeen different donors (10 ♂, 7 ♀) were used for all experiments. The average age was 66 ± 9.4 years. Cells were not pooled; one donor was used for one scaffold per experiment.

### 2.3. Test Setup

Titanium scaffolds were placed in standard six well culture plates and saturated with medium to avoid air bubbles within the pores. After the pre-cultivation, osteoblasts in the second passage were detached from the flask bottom using trypsin/EDTA (Gibco^®^-Invitrogen, Darmstadt, Germany) after being rinsed with phosphate buffered solution (PBS) (PAA, Coelbe, Germany). Cell suspension was adjusted to 4 × 10^5^ cells in 100 µL per scaffold and homogenously distributed onto the scaffold surface in 10 µL drops. After cells adhered for 30 min, medium was added until the scaffolds were completely covered. Static cultivation was done over eight days with medium changes after two, four and seven days.

**Figure 3:**
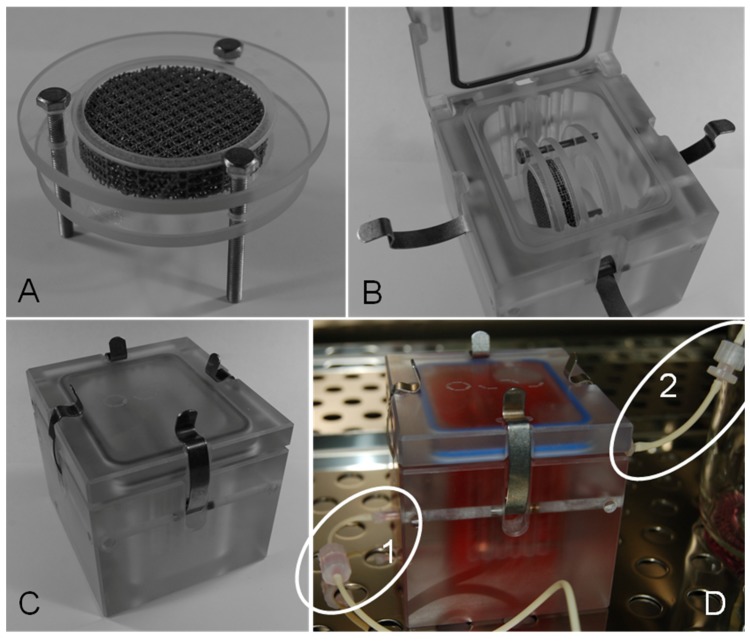
Scaffold placement. (**A**) Titanium scaffold fixed in polycarbonate disks; (**B**) Bioreactor with inserted scaffold; (**C**) Closed bioreactor. (**D**) Dynamic cultivation of a scaffold placed in the closed per(i)fusion system. 1D—medium influx. 2D—medium efflux.

**Figure 4:**
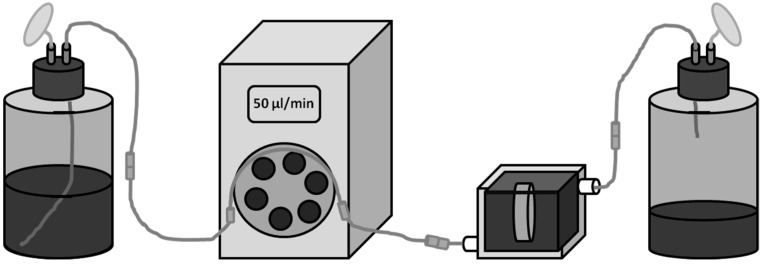
Test system. From left to right: Cooled medium reservoir with air filter, ISMATEC peristaltic pump, reactor with scaffold, waste container. Connections via gas permeable silicon tubes and luer-lock pins.

For dynamic cultivation, cells were allowed to adhere for 24 h on a scaffold in static culture, which then was transferred to a per(i)fusion-chamber (Minucells and Minutissue Vertriebs GmbH, Bad Abbach, Germany) under sterile conditions. Thereby, the scaffold was placed in specific retainers made of polycarbonate and were placed horizontally in the medium-filled reactor (Figure 3A,B). The system (containing of bioreactor and hose connections) was refilled with additional medium and was hermetically closed via gasket and clamps (Figure 3C). The cell seeded surface was orientated towards the fresh medium influx. Gas permeable silicon tubes for CO_2_ exchange (PharMed^®^ BPT, ID 0.25 mm, Ismatec, IDEX Health & Science GmbH, Wertheim, Germany) and luer-lock pins enabled connection to a shaded and constantly cooled medium reservoir flask and a waste flask (Figure 3D). By means of a peristaltic pump (MCP, Ismatec, IDEX Health & Science GmbH, Wertheim, Germany) a constant upwards medium flow rate (50 µL/min) through the chamber was maintained. This flow rate is deduced from previous works of this research group [39]. The complete system is illustrated in Figure 4. The appropriate level of shear stress cannot be elevated in this system. Cells were cultured for further 72 h in the dynamic system and then the scaffold was transferred back to a 6-well plate for analyzing.

### 2.4. Cell Biological Tests

Analyses of cell viability and migration were carried out after one, four and eight days to evaluate chronological sequence of cell behavior. The colorimetric WST-1 assay was conducted to examine cell viability on the four different types of titanium scaffolds. The tetrazolium salt WST is transformed to formazan by mitochondrial succinate dehydrogenase of metabolic active cells. The adsorption is measured at 450 nm in a microplate reader (Opsys MRTM, Dynex Technologies GmbH, Denkendorf, Germany) and is directly proportional to the metabolic cell activity. Qualitative cell viability was analyzed by means of live/dead staining with the two fluorescence dyes calcein AM for vital cells and ethidium homodimer-1 for dead ones (Live/Dead Cell Viability Assay, Invitrogen, Darmstadt, Germany). An enzyme linked immunosorbent assay (Metra C1CP EIA Kit, Quidel, Buende, Germany) was done to verify synthesis of pro-collagen type 1 by the osteoblasts after static culture. A detection of collagen in the cell supernatant while or after dynamic cultivation was not possible due to the reactor construction and continuous medium flow.

### 2.5. Statistical Analysis

The statistical significance of the influence of the manufacturing processes comparing the cubic EBM and SLM scaffolds was evaluated by ANOVA Post-Hoc-LSD using IBM^®^ SPSS^®^ Statistics Version 20 (IBM Corp., New York, NY, USA). Furthermore, the structural influence was assessed between the SLM scaffolds with cubic, pyramidal and diagonal designs. The significance level was set to *p* < 0.05.

## 3. Results

### 3.1. Cubic Structure—Influence of the Manufacturing Method

**Figure 5:**
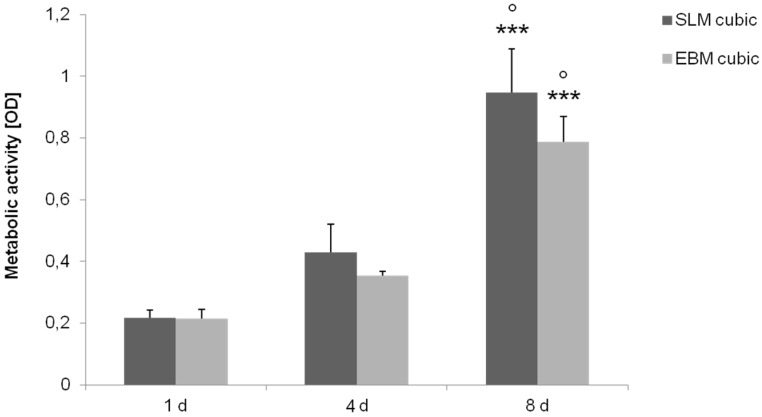
Metabolic activity of human osteoblasts cultured under static conditions over eight days on cubic structures (SLM, EBM). Values are means ± SD (*n* ≥ 3). ANOVA Post-Hoc-LSD: Statistical significance levels (° *p* < 0.05, *** *p* ≤ 0.001) compared to the respective four days (°) and one day (*). No significant differences revealed between the several manufacturing methods.

Titanium scaffolds with cubic microstructure (pore size 700 µm, porosity ~51%) manufactured with EBM and SLM were seeded with human osteoblasts and cultured under static and dynamic conditions. Static cultivation showed no significant differences of metabolic cell activity between the manufacturing methods investigated over time, but revealed a significant increase for both structures in general after eight days (*p* ≤ 0.001) (Figure 5).

**Figure 6:**
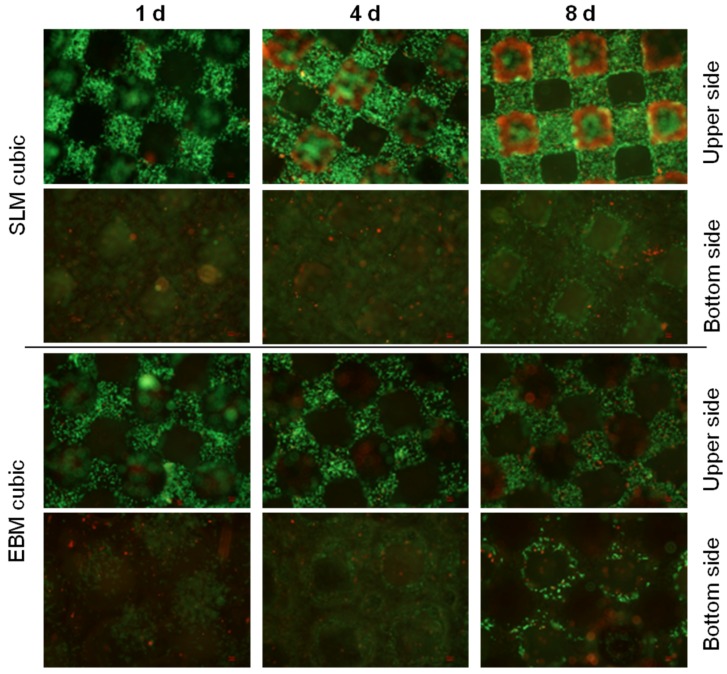
Live/dead staining of cubic scaffolds after static cultivation (one day, four days and eight days). Living cells are displayed in green, dead ones in red. Magnification: 40×. Scale bar: 50 µm (down right).

Live/dead staining illustrated an increase of living (green), but also dead cells (red) as well as higher cell density over time (Figure 6). Furthermore, living cells in lower layers are mainly visible at the pyramidal and the diagonal structured scaffolds. After eight days migrated cells can only be assumed in lower layers, but seem to have died off lacking supply with oxygen and nutrients.

**Figure 7:**
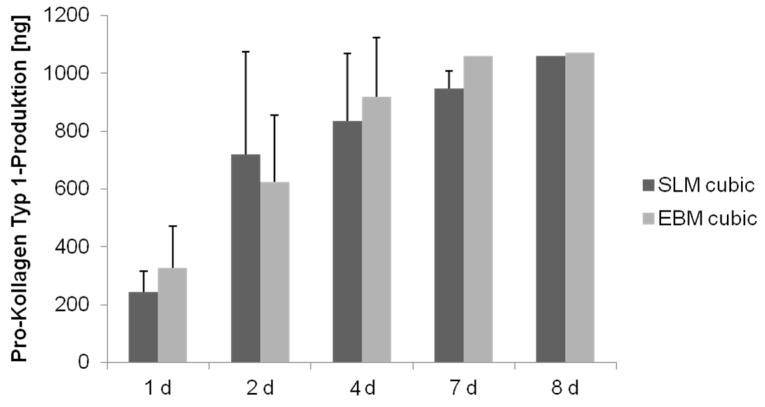
Collagen synthesis of human osteoblasts cultured under static conditions over eight days. Values are means ± SD. (*n* ≥ 1): ANOVA Post-Hoc-LSD. Statistical significance levels (*p* < 0.05).

Due to the design of the bioreactor, measurement of collagen synthesis could only be done after static cultivation. Detection of collagen type 1 production revealed a significant increase over time for both cubic scaffolds (SLM: *p* < 0.008; EBM: *p* < 0.019), but no significant difference between, confirming a missing influence of the manufacturing method on protein synthesis (Figure 7).

**Figure 8:**
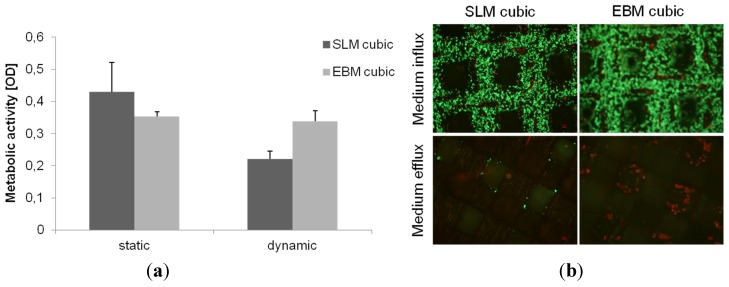
(**a**) Metabolic activity of human osteoblasts cultured on cubic scaffold designs under dynamic and static conditions after four days of cultivation. Values are means ± SD (*n* ≥ 3). Statistic revealed no significant differences between static and dynamic cultivation. (**b**) Live/dead staining of cubic scaffolds after dynamic cultivation after total five days. Living cells are displayed in green, dead ones in red. Magnification: 40×. Scale bar: 50 µm (down right).

In case of dynamic cultivation the cubic scaffolds were placed in a bioreactor after 24 h static pre-cultivation and cultured for further three days. Human osteoblasts on the SLM and EBM manufactured scaffolds exhibited no significant increased metabolic activity after dynamic cultivation compared to the static one (Figure 8). Comparison between both types of scaffolds after dynamic cultivation showed also no significant differences.

Cells on the upper side of the scaffolds showed dense cell collectives, but cell migration to the bottom side of the scaffold can only be observed for the EBM scaffold detecting dead cells (Figure 8).

### 3.2. Fabrication via Selective Laser-Melting—Comparison of Different Structures

**Figure 9:**
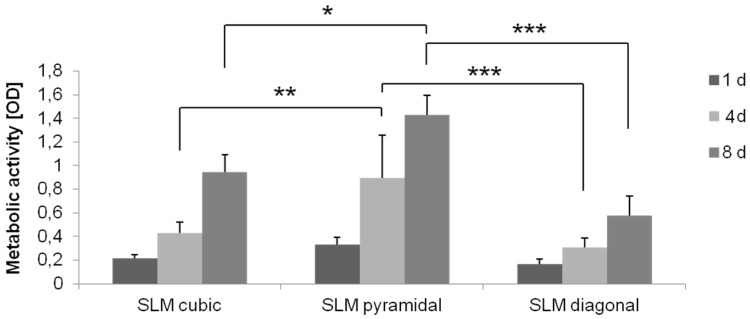
Metabolic activity of human osteoblasts cultured under static conditions over eight days on SLM manufactured structures with several designs (cubic, pyramidal, diagonal). Values are means ± SD (*n* ≥ 3). ANOVA Post-Hoc-LSD: Statistical significance levels (* *p* < 0.05, ** *p* < 0.01, *** *p* < 0.001).

Based on the results of the comparison between the two manufacturing methods and the biomechanical investigations [5,37], the selective-laser melting was used for further structures to compare. Titanium scaffolds manufactured via SLM with three different microstructures (cubic, pyramidal, diagonal) were seeded with human osteoblasts and cultured under static and dynamic conditions. Static cultivation showed a significant higher metabolic cell activity on the pyramidal scaffold compared to the other ones after four and eight days (cubic *vs.* pyramidal: *p* = 0.002, *p* = 0.013; diagonal *vs.* pyramidal: *p* ≤ 0.001, *p* ≤ 0.001). No significant differences could be determined between the cubic and diagonal structure at any point. In general, a significant increase of activity was detected for all structures over time (all: *p* ≤ 0.001) (Figure 9).

**Figure 10:**
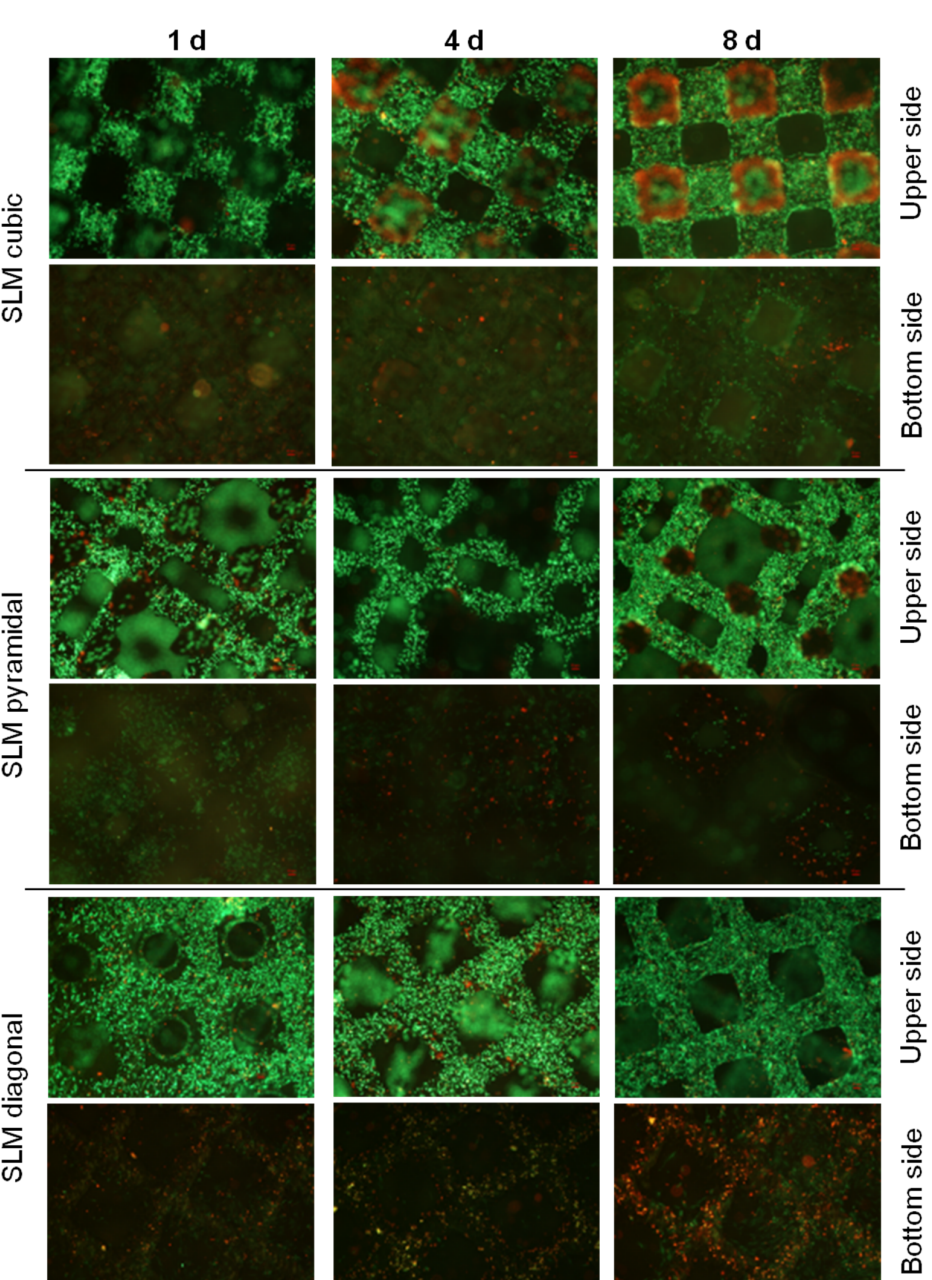
Live/dead staining of SLM manufactured scaffolds with several designs after static cultivation (one day, four days and eight days). Living cells are displayed in green, dead ones in red. Magnification: 40×. Scale bar: 50 µm (down right).

Live/dead staining illustrated an increase of living (green), but also dead cells (red) as well as higher cell density over time (Figure 10) and especially, living cells in lower layers for the diagonal and pyramidal structures. Over time migrated cells can mainly be observed in lower layers of the pyramidal and diagonal structures, but not in the cubic one, possibly due to lacking supply with oxygen and nutrients.

**Figure 11:**
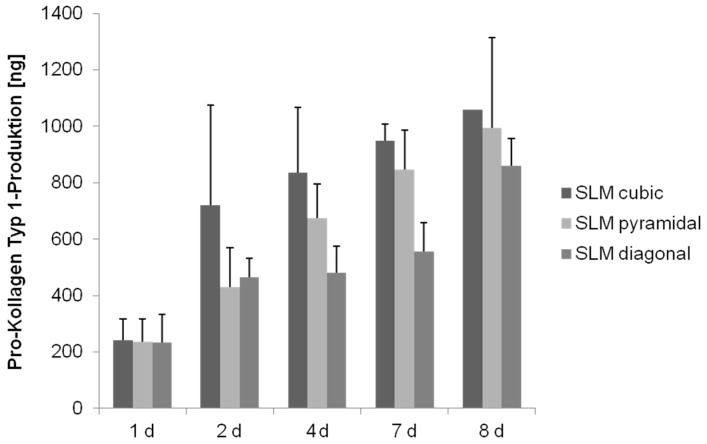
Collagen synthesis of human osteoblasts cultured under static conditions over eight days. Values are means ± SD. (*n* ≥ 1): ANOVA Post-Hoc-LSD. Statistical significance levels (*p* < 0.05).

Detection of collagen type 1 production revealed a significant increase over time for all three scaffold structures (cubic: *p* < 0.008; pyramidal: *p* = 0.002; diagonal: *p* = 0.001), but a significant lower synthesis on the diagonal one over time (*p* ≤ 0.008) (Figure 11).

**Figure 12:**
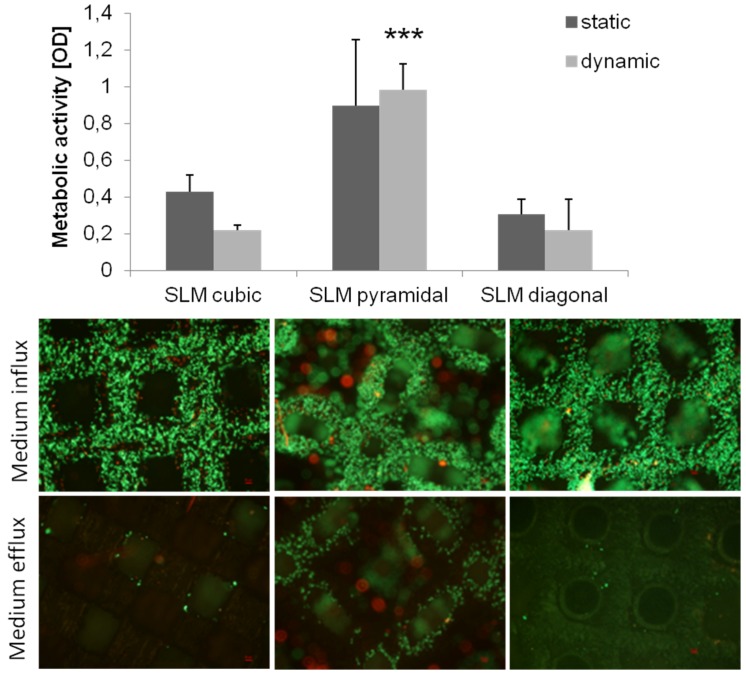
Metabolic activity of human osteoblasts cultured on SLM manufactured scaffold with several designs under dynamic and static conditions after four days of cultivation. Values are means ± SD (*n* ≥ 3). ANOVA Post-Hoc-LSD: Statistical significance levels (*** *p* < 0.001) compared to the further designs after dynamic cultivation. Statistic revealed no significant differences between static and dynamic cultivation (above). Live/dead staining of SLM manufactured scaffolds after dynamic cultivation after four days. Living cells are displayed in green, dead ones in red. Magnification: 40×. Scale bar: 50 µm (down right) (below).

Dynamic cultivation was done as described previously. Human osteoblasts on the SLM manufactured scaffold structures revealed no significant differences between the cultivation methods for all structures, but showed a significant higher activity on the pyramidal scaffold (*p* < 0.001) after dynamic cultivation compared to the other structures (Figure 12).

Live/dead staining illustrated a high cell density after dynamic cultivation, partly in lower layers of the scaffolds (Figure 12). The pyramidal structure with the highest metabolic activity also exhibits living cells at the side of medium efflux as well as ones in overlying layers, hence a cell migration through the scaffold without cell dying can be assumed.

## 4. Discussion

Synthetic, open-porous three-dimensional scaffolds are used for the treatment of large bone defects in consequence of trauma, tumors, tissue degeneration or congenital deformation [30]. In our study, we compared four different types of titanium scaffolds concerning the colonization with human osteoblasts during cultivation under static and dynamic conditions. First of all, we showed that titanium is an adequate material for three-dimensional scaffold fabrication by AM processes and for cellular biocompatibility as previously described [5,40].

### 4.1. Static Cultivation

During static cell cultivation, no significant differences in metabolic activity between the different manufactured scaffolds (SLM, EBM) were found over time. Hence, no distinct predominance of one manufacturing method could be found regarding the cubic structures, which was previously described [16]. However, selective-laser-melting revealed a more accurate and regular geometry of pores and struts (Figure 2F). Surface roughness by all means enhances osteoblastic adhesion and proliferation as pointed out by Karageorgiou and Kaplan [9]. Ingrowth in rough and porous structures is favored by osteoblasts [41]. The illustrated surfaces of the tested titanium scaffolds (Figure 2E–H) allude to different surface roughness with a possible influence on cell adhesion. Though, a higher roughness of the electron beam-melted titanium can be assumed and could be proven in exemplary tests in our research group, preferred by osteoblasts to adhere, actually [41]. Static cultivation of the SLM manufactured structures (cubic, pyramidal, diagonal) revealed no significant differences in metabolic cell activity after one day. With increasing cultivation duration, however, cellular activity of the human osteoblasts significantly increased on the SLM manufactured scaffold with pyramidal basic structure after 96 h and eight days. In contrast, at none of the other time periods significant differences occurred between the two other structures. An increasing proliferation and cell spreading was proven for a similar period by Chen *et al.* [12] and also up to 14 days by Hollander *et al.* [42]. Furthermore, in this work the scaffold with the highest porosity (76%) and smallest pores (400 to 620 µm) enabled the highest metabolic activity and ingrowth of the human osteoblasts over time. Smaller pores are proved to enhance cell proliferation and migration due to a higher surface for initial cell adhesion and relieved cell bridging [12,20,24]. However, a hundred percent plugging of the voids is designated for pores with 450–500 µm, [10] but was not detected on our scaffolds. At the top layer of the scaffolds used in our *in vitro* tests, growth of human cells was clearly cognizable along the struts for every structure, as indicated for increasing pore sizes [10]. High porosities up to 75% as used for the pyramidal and diagonal structures are referred to be necessary for sufficient permeability and cause Young’s modulus and compressive strength comparable to cancellous bone [23]. Advantage to the diagonal structure with similar values of porosity and pore size might be due to its basic structure narrowing the pore void and thereby inhibiting oxygen, nutrient and waste transport as well as cell migration. Furthermore, the mechanical stiffness might have influence on growth and matrix synthesis since osteoblasts are mechano-sensitive. The appropriate mechanical tests are published by Wieding *et al.* [43]. The slightly increased metabolic activity on the cubic structures compared to the diagonal one results from the lower porosity and four times wider struts leading to a larger surface area for initial adhesion [20]. The increase of dead cells in lower layers (see Figure 6 and Figure 10) and absent of cell migration during static cultivation is due to the initially seeded dense and thick cell layers on the top layer clogging the underlying pores [4]. With a lack of a supplemental continuous medium flow, nutrient supply is inhibited and, thereby, acidification of media in the scaffold centre and deficient provision with oxygen promoted [16,44].

We detected an increase of collagen production over time during cultivation under static conditions for the structures as well as manufacturing methods, significantly lower in the diagonal structured scaffold due to its basic structure, presumably, as mentioned above. Contrary to the expectations, collagen synthesis of cells on the pyramidal scaffold was not significantly higher compared to the other structures. Hence, as previously described, collagen synthesis is not influenced by the scaffold’s pore size [45].

### 4.2. Dynamic Cultivation

In our present study, dynamic cultivation supported an increased cell activity as well as migration through the pyramidal scaffold. Possible reasons were mentioned above considering porosity, pore size and basic scaffold structure. An adequate medium flow through the pyramidal structured scaffold can be assumed due to the high porosity and the open basic structure [23]. McCoy *et al.* [46] stated that pore size had no effect on the shear stress transferred to the cells by fluid flow. In contrast to previous studies [28,44], we could not confirm improved bone cell activity after dynamic cultivation compared to static conditions testing all four scaffold designs. Nevertheless, enhanced cell migration due to flow conditions was demonstrated in this work and is also proven by others [35,47]. So, a beneficial influence of our bioreactor is partially given depending on the chosen structure, but could be improved with appropriate adaptations and modifications, mentioned below. Several *in vitro* studies [27,28,32,48] were carried out with perfusion systems using flow rate intensities from 0.01 mL/min to 4 mL/min, mainly in excess of 0.1 mL/min. Overall, possibly the flow rate of 50 µL/min (=0.05 mL/min) was chosen too low in that case, but is similar to Jaasma *et al.* (0.05 mL/min) [49]. The flow rate and frequency influences the cell response and cause cell detachment and necrosis at too high levels [28]. In oscillatory or pulsatile perfusion systems, rather according to physiological fluid flow profiles [50], flow rates even up to 10 mL/min were used [30]. Osteoblasts are shown to be more mechano-responsive to those forms of perfusion as to steady one [49,50]. It must be pointed out that velocities and shear rates of the culture medium may differ in several zones of the scaffold, for instance those might be higher in the boundary area. In case of that, cell response will slightly differ in the appropriate areas. That should be simulated and determined for further studies.

In future studies an adaptation and a variation of the flow rate and cultivation period can be conducted in order to assess the influence of flow rate intensity and of time to cell behavior on the titanium scaffolds. Furthermore, a novel perfusion system will be tested, to enforce the medium to flow through the scaffold. Volkmer *et al.* [51] modified the same model of bioreactor used in this work from a per(i)fusion to perfusion system avoiding that medium flows around the scaffold promoting an enhanced oxygen supply. Moreover, cell growth on and into the scaffold is limited by its height and width [27]. In addition, evaluation of broader parameters will be necessary to understand the impact of the structural conditions used. For instance, protein expression as well as mRNA analysis would help to identify the osteogenic phenotype of the cells. So far, RNA isolation from the titanium scaffolds proved to be difficult and afforded to low yields for further steps like qRT-PCR. Thus, protein-analysis via ELISA while or after dynamic cultivation was constricted by the current reactor construction and a too heavily diluted supernatant in the waste container, moreover, containing synthesized proteins from the whole test duration making a defined evaluation for one time point difficult. Additionally, levels of alkaline phosphatase, osteocalcin and intracellular calcium could be detected [35]. As previously done by other groups [26,51], evaluation of oxygen amount and pH-value seems to be a needful control for supply in the scaffold centre while dynamic cultivation.

## 5. Conclusions

Three-dimensional open-porous titanium scaffolds of two manufacturing methods with three different structural designs, porosities and pore sizes were examined as most suitable structure for human osteoblasts under static and dynamic conditions. Titanium proved to be an adequate scaffold material for *in vitro* cell tests targeting to bridge large bone defects *in vivo*. Moreover, at choice of an additive manufacturing method selective-laser-melting could be preferred as suitable generative process with more defined structure borders. This study could not point out a cultivation method (static *vs.* dynamic) enhancing the cellular proliferation more than the other one using the per(i)fusion flow bioreactor at 50 micro litres per mL. However, dynamic cultivation resulted in an improved cell migration through the porous titanium scaffold. Altogether, the structure, porosity and pore size of a scaffold influences cell growth behavior. The scaffold with smaller pores (400 to 620 µm), a high porosity (75%) and an open-porous pyramidal basic structure enabled the highest metabolic cell activity and migration and is proved be the most suitable leading structure so far. This has to be verified in further animal tests.
